# Chemotherapy without anthracyclines for acute promyelocytic leukemia in a 5‐year‐old male patient with Fontan procedure

**DOI:** 10.1111/ped.70083

**Published:** 2025-06-03

**Authors:** Hidehiro Minegishi, Atsushi Makimoto, Motohiro Matsui, Yuichi Yokokawa, Jun Maeda, Yuki Yuza

**Affiliations:** ^1^ Department of Hematology/Oncology Tokyo Metropolitan Children's Medical Center Tokyo Japan; ^2^ Department of Cardiology Tokyo Metropolitan Children's Medical Center Tokyo Japan

**Keywords:** APL, ATO, ATRA, Fontan, HLHS

The prognosis of acute promyelocytic leukemia (APL) has improved with the advent of differentiation therapy based on all‐trans retinoic acid (ATRA). Anthracycline‐based chemotherapy, which is cardiotoxic, leads to complete remission in more than 90% of newly diagnosed APL cases when combined with ATRA.^1^

Hypoplastic left heart syndrome (HLHS) is a type of congenital heart disease with a poor prognosis. Multiple surgeries to create Fontan circulation are required for long‐term survival.

We present a 5‐year‐old male patient with APL and Fontan circulation who received arsenic trioxide (ATO) and ATRA without anthracyclines following the approval of the regimen by the hospital ethics committee and with the informed consent of his guardian.

The patient received a diagnosis of HLHS at birth and underwent Norwood surgery, atrial septal defect creation, and Blalock‐Taussig shunt surgery at the age of 2 months. He underwent bidirectional Glenn procedure at the age of 11 months and Fontan surgery at the age of 2 years. At the age of 5 years, he was admitted for pancytopenia and blasts in the peripheral blood. A blood test revealed WBC 1.05 × 10^9^/L and blast cells 17.0%. A bone marrow (BM) smear revealed pathological promyelocytes accounting for 82% of the BM cells and having cytoplasm with abundant, coarse, azurophilic granules and Auer bodies. Immunophenotyping of the abnormal cells found the expression of CD13, CD33, CD64, CD117, and MPO. A *PML*::*RARA* fusion gene was detected by RT‐PCR. No FLT3‐ITD mutation was found.

He received a diagnosis of APL and began receiving ATRA 25 mg/m^2^ with dexamethasone 0.4 mg/kg to prevent APL differentiation syndrome on hospital day 1. Intravenous trioxide (ATO) 0.15 mg/kg was begun on day 10, based on a report of a case series of pediatric APL from the German BFM group.^2^ However, the administration of ATO was stopped on day 13 because the WBC count rose above 30 × 10^9^/L (Figure 1). As the WBC count increased, the patient developed dyspnea, pleural effusion, and weight gain, symptoms related to differentiation syndrome, and oxygen administration was started. When the WBC count exceeded 60 × 10^9^/L on day 17, intravenous cytarabine 40 mg/m^2^ was begun for cytoreduction. After the WBC count decreased and cytoreduction was completed on day 20 at WBC 34.12 × 10^9^/L, ATO administration was resumed on day 21 at WBC 16.74 × 10^9^/L. ATRA and ATO were administered as induction therapy until day 42 after the BM morphology and molecular complete remission. As the white blood cell count improved, the symptoms associated with differentiation syndrome also improved, and oxygen administration was discontinued after induction therapy. Echocardiography demonstrated no change in cardiac function before and after induction therapy.

**FIGURE 1:**
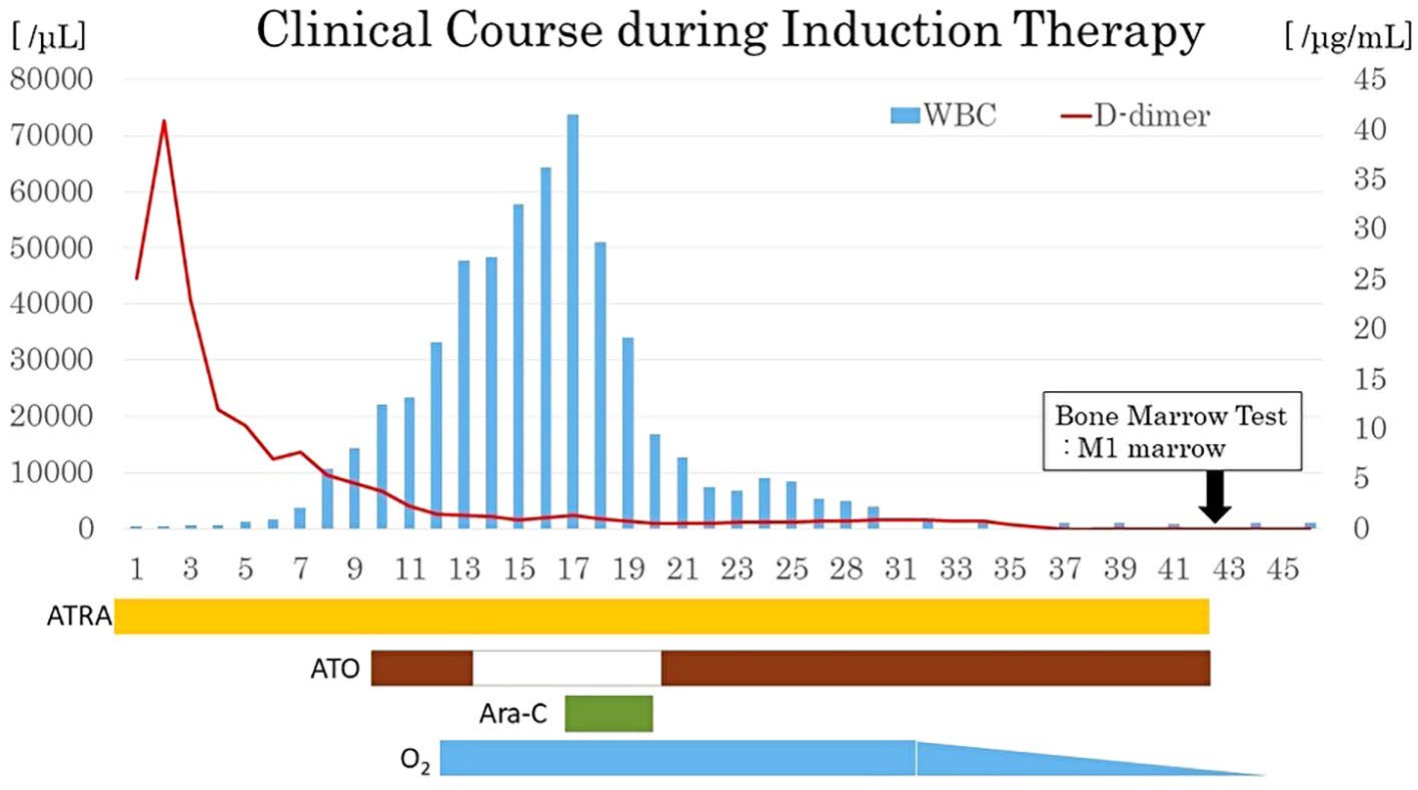
Clinical course during induction therapy.

After a two‐week rest, the patient began four cycles of maintenance therapy consisting of ATRA 25 mg/m^2^ on days 1–14 and 29–42, ATO 0.15 mg/kg on days 1–28, and intrathecal therapy consisting of cytarabine 40 mg/m^2^ on day 1. The duration of maintenance therapy was 32 weeks with 8 weeks per cycle, during which no serious complications were observed. No recurrence has been detected more than 1 year after the completion of maintenance therapy.

A previous, retrospective case series reported a high remission rate associated with ATO + ATRA treatment alone in pediatric patients.^2^ The results of a randomized, phase‐3 trial involving induction therapy and intensive therapy with ATRA + ATO demonstrated a higher, four‐year, molecular‐genetic remission rate than ATRA or idarubicin alone in adult patients with APL.^3^ Large, prospective clinical trials of ATRA + ATO as well as of oral arsenic enrolling 193 pediatric patients with APL in China found that the two‐year OS rate was 99% (95% confidence interval [CI]: 97–100) while the two‐year EFS rate was 97% (95% CI: 93–100) in a standard‐risk group with a WBC count <10 × 10^9^/L and no FLT3‐ITD mutation.^4^

Pediatric patients who have received anthracycline >300 mg/m^2^ are associated with a significantly higher incidence of heart failure. A cumulative anthracycline dosage >100 mg/m^2^ can decrease left ventricular fractional shortening and increase the afterload.^5^ Anthracycline administration may precipitate heart failure in pediatric patients with heart disease.

## AUTHOR CONTRIBUTIONS

HM drafted the original manuscript. YY (Yuki Yuza) made substantial contributions to the conception of the work. AM, MM, YY (Yuichi Yokokawa) and JM substantially contributed to the revision of the manuscript drafts. All authors have approved the submitted version of the manuscript and agreed to be accountable for any part of the work.

## CONFLICT OF INTEREST STATEMENT

The authors declare no conflict of interest.
